# Multiple Pipeline Embolization Devices for the Treatment of Complex Intracranial Aneurysm: A Multi-Center Study

**DOI:** 10.3389/fnagi.2022.905224

**Published:** 2022-06-13

**Authors:** Feng Fan, Yu Fu, Jianmin Liu, Xinjian Yang, Hongqi Zhang, Tianxiao Li, Huaizhang Shi, Jieqing Wan, Yuanli Zhao, Yunyan Wang, Wenfeng Feng, Donglei Song, Yang Wang, Guohua Mao, Aisha Maimaitili, Sheng Guan

**Affiliations:** ^1^Department of Neurointervention Radiology, The First Affiliated Hospital of Zhengzhou University, Zhengzhou, China; ^2^Changhai Hospital Affiliated to Naval Medical University, Shanghai, China; ^3^Department of Interventional Neuroradiology, Beijing Neurosurgical Institute, Beijing Tiantan Hospital, Capital Medical University, Beijing, China; ^4^Department of Neurosurgery, Xuanwu Hospital, Capital Medical University, Beijing, China; ^5^Zhengzhou University People’s Hospital, Zhengzhou, China; ^6^First Affiliated Hospital of Harbin Medical University, Harbin, China; ^7^Renji Hospital, School of Medical, Shanghai Jiao Tong University, Shanghai, China; ^8^Peking University International Hospital, Beijing, China; ^9^Qilu Hospital of Shandong University, Jinan, China; ^10^Nanfang Hospital, Southern Medical University, Guangzhou, China; ^11^Shanghai Donglei Brain Hospital, Shanghai, China; ^12^First Affiliated Hospital of Nanchang University, Nanchang, China; ^13^Second Affiliated Hospital of Nanchang University, Nanchang, China; ^14^First Affiliated Hospital of Xinjiang Medical University, Ürümqi, China

**Keywords:** anterior circulation, giant intracranial aneurysm, pipeline embolization device, posterior circulation, salvage therapy

## Abstract

**Background:**

The Pipeline for Uncoilable or Failed Aneurysms (PUFS) trial primarily demonstrated the safety and efficacy of the implantation of multiple pipeline embolization devices (multi-PEDs) for large/giant intracranial aneurysms. However, no study has focused on when, why, or how to apply multi-PEDs.

**Objective:**

The purpose of this study was to investigate the indications and strategies of using multi-PEDs for complex intracranial aneurysms.

**Methods:**

Patients who had been treated with two or more PEDs were included in the post-market multicenter registry study from 2014 to 2019, across 14 centers in China. Indications, strategies, perioperative safety, and clinical outcomes were retrospectively analyzed. The modified Rankin scale (mRS) score was used to evaluate clinical outcomes comprehensively, and the O’Kelly–Marotta (OKM) grading scale was used to evaluate aneurysm healing results.

**Results:**

A total of 55 intracranial aneurysms were treated with multi-PEDs. There were 20 fusiform aneurysms with a large range, 25 large/giant saccular aneurysms, six aneurysms with failed treatment, and four aneurysms with greatly varied diameters of the parent artery. The strategies included telescope techniques in 40 patients and overlap techniques in 15 patients. In total, 120 stents were deployed in 55 patients. The operation styles included 25 patients (55.6%) with two PEDs, 21 patients (38.2%) with two PEDs combined with coiling, four patients (7.3%) with three PEDs, four patients (7.3%) with three PEDs combined with coiling, and one patient (1.8%) with four PEDs. Angiography revealed OKM D in two, OKM C in seven, and OKM A and B in 46 cases after surgery. During the perioperative period, eight patients developed neurological dysfunction, three of whom died. A total of thirty-four patients were followed up with digital subtraction angiography for 2–45 (8.2 ± 8.0) months. Angiography revealed OKM D in 26, OKM C in five, and OKM B in three. At the last follow-up, the mRS score was 0–1 in 52 patients.

**Conclusion:**

The treatment of anterior circulation aneurysms with multi-PEDs is safe and effective. The implantation of multi-PEDs could be considered for large-scale fusiform aneurysms, large/giant saccular aneurysms with a jet-sign, salvage of failed PED treatments, and in cases where the diameter of the parent artery varies greatly.

## Introduction

The pipeline embolization device (PED) was approved by the Food and Drug Administration (FDA) in 2011 for the treatment of large and giant wide-neck intracranial aneurysms in the internal carotid artery. The PED for the intracranial treatment of aneurysms trial demonstrated significant advancements in aneurysm treatments (Nelson et al., 2011). Recently, an increasing number of cerebrovascular centers have considered PED as the preferred choice for unruptured complex aneurysms (Crobeddu et al., 2013). However, whether two or more PEDs are better for particular aneurysms remains controversial (Mona et al., 2013). This problem has potential effects on treatment strategies, safety, efficacy, and cost (El-Chalouhi et al., 2014). Thus, we here performed a retrospective analysis to explore the indications and strategies for the application of multiple pipeline embolization devices (multi-PEDs) for intracranial aneurysms.

## Materials and Methods

### Study Design and Patient Population

This was a multicenter, retrospective study performed in China. All patients in this study were selected from the PLUS registry study (ClinicalTrials.gov identifier: NCT03831672), which included those treated with PEDs for ruptured and unruptured intracranial aneurysms from November 2014 to October 2019. We enrolled 1,171 patients with 1,322 aneurysms from 14 medical centers. Our subgroup study aimed to investigate the indications and strategies for using multi-PEDs for complex intracranial aneurysms.

The inclusion criteria were as follows: (1) intracranial aneurysms treated with two or more PEDs or (2) salvage for failed PED treatment. Exclusion criteria comprised of (1) two or more PEDs used to treat aneurysms in different arteries and (2) subarachnoid hemorrhage (SAH).

### Antiplatelet Procedure

Patients were treated with aspirin (100 mg daily) and clopidogrel (75 mg daily) for 5–7 days before the operation. The aspirin and clopidogrel doses were adjusted preoperatively after platelet function testing. Platelet function tests were performed on all patients using thromboelastography. The platelet inhibition rates induced by arachidonic acid (AA) and adenosine diphosphate (ADP) were measured. Aspirin was considered effective if the inhibition rate of AA was ≥ 50%, and aspirin insensitivity was defined if it is < 50%. Patients insensitive to aspirin insisted or increased the dose appropriately. Clopidogrel was considered effective if the inhibition rate of ADP was ≥ 30%, and clopidogrel resistance was defined if it was < 30%. Patients insensitive to clopidogrel were administered ticagrelor (90 mg, two times daily). Dual antiplatelet therapy was continued for 3–6 months postoperatively. Individualized implementation was adjusted according to both the clinical syndromes and follow-up images.

### Procedural Technique

All patients were treated under general anesthesia *via* a transfemoral arterial approach. After standard angiographic projection, intra-arterial 3D rotational angiography was performed in all patients to determine the size and optimal working position, through 3D reconstruction and PED simulation procedures. A 7-F sheath (Shuttle Sheath, Cook Medical, Bloomington, IN, United States) was used to guide the multifunctional 5-F catheter (Medtronic, Minneapolis, MN, United States) and 0.035-inch super-slippery microguidewire (CHIKAI, Asahi Intecc, Nagoya, Japan) to the initial segment of the internal carotid artery or the first segment of the vertebral artery. Under the working position, a 5-F distal support catheter (Navien, 115 or 125 cm, Covidien, Medtronic) was inserted near the aneurysm neck. Assisted by the microcatheter, the microguidewire, and the 5-F distal support catheter, the stent catheter (Marksman, Covidien) was passed through the aneurysm neck and was placed at the distal segment of the parent artery. If the coil was indispensable, the embolization microcatheter was positioned through the 7-F guiding sheath, in parallel to the 5-F distal support catheter, and the head part of the microcatheter was introduced into the sac with the assistance of a 0.014-inch microguidewire. Subsequently, a multi-PED system was implanted. These strategies included telescope and overlap techniques.

Angiography in the working position was performed to clarify the retention of contrast and patency of the parent artery and distal branches. Flat-panel intra-arterial rotational angiography (VasoCT, Philips Healthcare, Best, Netherlands) or dynamic computed tomography angiography (DynaCT, Siemens Healthcare, Forchheim, Germany) was performed to assess device deployment. If necessary, post-operative adjustment could be applied to correct poor opening or wall attachment. X per-CT scan was performed to evaluate cerebral hemorrhage or SAH.

### Study Endpoints

Procedural success was defined as follows: (1) PED deployment with complete coverage of the aneurysm neck and good wall attachment, and (2) patency of the parent artery and distal branches. Angiographic and clinical follow-ups were performed at 3, 6, and 12 months and annually thereafter. The primary angiographic endpoint was complete aneurysm occlusion with no stenosis of the parent artery. The O’Kelly–Marotta (OKM) grading scale was used to evaluate aneurysm embolization (O’kelly et al., 2010). The stenosis of the parent artery was defined as a stenosis rate of > 50% on imaging (Kwolek et al., 2015). Clinical outcomes were assessed using the modified Rankin scale (mRS) (Wang et al., 2020). Major adverse cerebrovascular complications included transient ischemic attack, embolization in the new territory, and SAH.

## Results

### Baseline Patient and Aneurysm Characteristics

Among the 55 patients, 31 were men and 24 were women. Their ages ranged from 8 to 76 (46.7 ± 15.9) years. Among them, 49 patients presented with headache, visual disturbance, ischemia-related symptoms, or other symptoms, and six cases presented with accidental findings. All patients underwent digital subtraction angiography (DSA) before endovascular treatment. A total of thirty-nine aneurysms were located in the internal carotid arteries, 13 in the vertebrobasilar arteries, two in the middle cerebral arteries, and one in the posterior cerebral artery. The maximum diameter was 4.1–51.1 (24.2 ± 14.1) mm, and the morphological aneurysm classifications included 30 sacs, 21 fusiform, and four irregular sacs (Table 1).

**TABLE 1:** Baseline patient and aneurysm characteristics.

	Study population
Age, year	46.7 ± 15.9
Male sex	31 (56.4%)
**Morphology**	
Saccular	30 (54.5%)
Fusiform*	21 (38.2%)
Irregularity	4 (7.3%)
**Location**	
ICA	39 (70.9%)
MCA	2 (3.6%)
VA	8 (14.5%)
VB	3 (5.5%)
BA	2 (3.6%)
PCA	1 (1.8%)
**Size (Maximum diameter)**	
< 10 mm	6 (10.9%)
10 mm–25 mm	28 (50.9%)
> 25 mm	21 (38.2%)

*Data are showm as n (%) or the mean ± SD. Fusiform includes fusiform aneurysms, snakelike aneurysms and dissection aneurysms involving a large range. ICA, Internal carotid artery; MCA, Middle cerebral artery; VA, Intracranial segment of vertebral artery; VB, Both vertebral and basilar arteries were involved; BA, Basilar artery; PCA, Posterior cerebral artery.*

### Operative Results

Multiple pipeline embolization devices were successfully implanted in 55 patients. These cases included 20 fusiform aneurysms with a large range, 25 large/giant saccular aneurysms, six aneurysms with failed treatment, and four aneurysms in which the diameters of the parent artery varied greatly. The strategies included telescope techniques in 40 patients and overlap techniques in 15 patients. In total, 120 PEDs were deployed in 55 patients. The operation styles included 25 patients (55.6%) with two PEDs, 21 patients (38.2%) with two PEDs combined with coiling, four patients (7.3%) with three PEDs, four patients (7.3%) with three PEDs combined with coiling, and one patient (1.8%) with four PEDs. The PEDs were successfully opened in 53 patients and required adjustment in two patients. Angiography revealed OKM A and B in 46 patients, OKM C in seven patients, and OKM D in two patients (Table 2).

**TABLE 2:** Treatment details and results.

	Frequency
**PED type**	
PED Classic	31 (56.4%)
PED Flex	24 (43.6%)
**Reasons of Multi-PEDs**	
Fusiform with a large range	20 (36.4%)
Large/giant saccular aneurysms	25 (45.6%)
Salvage for failed treatment	6 (10.9%)
Diameters vary greatly	4 (7.3%)
**Operation Styles**	
PED + PED	25 (55.6%)
PED + PED + Coil	21 (38.2%)
PED + PED + PED	4 (7.3%)
PED + PED + PED + Coil	4 (7.3%)
PED + PED + PED + PED	1 (1.8%)
**Device deployment**	
Successful	53 (96.4%)
Successful after adjustment	2 (3.6%)
Unsuccessful	0
**Radiographic Result (perioperative period)**	
OKM A and B	46 (83.6%)
OKM C	7 (12.7%)
OKM D	2 (3.6%)
**Clinical Results (perioperative period)**	
mRS ≤ 2	51 (92.7%)
mRS > 2	4 (7.3%)
**Perioperative complications**	8 (14.5%)
TIA	3 (5.5%)
ENT	3 (5.5%)
SAH	2 (3.6%)

*Data are presented as n (%) or n (%). TIA, transient ischemic attack; ENT, embolization in the new territory; SAH, subarachnoid hemorrhage; OKM, O’Kelly–Marotta grading scale.*

During the perioperative period, eight patients developed neurological dysfunction (Table 3). A total of three patients died, one patient developed SAH and received conservative treatment (mRS 2), one patient developed post-operative hypoperfusion (mRS 4), one patient developed thrombogenesis during the operation (mRS 0), and two patients developed transient ischemic attacks (mRS 0-1). Among the three died patients, two were with brainstem compression and ischemic stroke of posterior circulation and one suffered with SAH of anterior circulation in the early post-operative period.

**TABLE 3:** Perioperative complications.

Case	Location	Operation styles	Complications	Treatment	mRS	
					Pre-operation	3∼10 days after operation
Case 1	ICA	PED + PED + Coil	SAH	Conservative treatment	1	2
Case 2	VB	PED + PED	ENT	Conservative treatment	0	6
Case 3	MCA	PED + PED	TIA	Conservative treatment	1	1
Case 4	ICA	PED + PED	SAH	Surgery	1	6
Case 5	ICA	PED + PED + Coil	TIA	Tirofiban	0	0
Case 6	VB	PED + PED + Coil	TIA	Conservative treatment	1	1
Case 7	ICA	PED + PED + PED	ENT	Conservative treatment	0	4
Case 8	VB	PED + PED	ENT	Conservative treatment	1	6

*TIA, transient ischemic attacks; ENT, embolization in new territory; SAH, subarachnoid hemorrhage.*

### Radiographic and Clinical Results

A total of thirty-four patients were followed up with DSA for 2–45 (8.2 ± 8.0) months. Angiography revealed complete occlusion in 26 patients (OKM D), near occlusion in five patients (OKM C), and the lack of occlusion in three patients (OKM B). During follow-up, one patient had internal carotid artery occlusion on the operation side (mRS 0), one patient had asymptomatic occlusion of the ophthalmic artery on the operation side (mRS 0), and one patient had transitory blindness (mRS 0). The remaining patients had no stenosis of the parent arteries and no new neurological complications. At the last follow-up, the mRS score was 0–1 in 52 patients (Table 4).

**TABLE 4:** Radiographic and clinical results.

	Frequency
**Clinical results**	
mRS ≤ 2	52/52 (100%)
mRS > 2	0
**Radiographic results**	
OKM A and B	3/34 (8.8%)
OKM C	5/34 (14.7%)
OKM D	26/34 (76.5%)
**Parent artery status**	
Patent	33/34 (97.1%)
Stenosis	0
Occluded	1/34 (2.9%)

*Data are shown as n (%), n/N (%).*

## Discussion

There is an increasing number of reports on the treatment of intracranial aneurysms with PED. However, few studies have focused on the application of multi-PEDs. This study included 55 complex aneurysms, which accounted for approximately 4.2% of all aneurysms (55/1322) at our institutions. A retrospective analysis was conducted to explore the safety, efficacy, indications, and strategies of multi-PED implantation. These aneurysms had the following characteristics: (1) fusiform aneurysms with a large range; (2) large/giant aneurysms with jet-sign after a PED implantation; (3) aneurysms with failed PED treatment, requiring salvage; and (4) aneurysms where the diameter of the parent artery varied greatly between the inflow and outflow vessels (> 2 mm). Our study demonstrated that multi-PED implantation is safe and effective for the treatment of complex intracranial aneurysms, yielding high occlusion and low complication rates.

### Indications for Multiple Pipeline Embolization Devices

**FIGURE 1:**
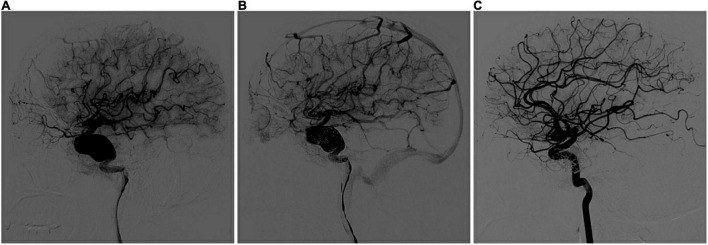
Multi-PEDs for the treatment of a fusiform aneurysm (telescope technique). **(A)** Digital subtraction angiography (DSA) identified a fusiform aneurysm with a proximal diameter of 4.87 mm and a distal diameter of 4.38 mm in the internal carotid artery before operation. **(B)** After three pipeline embolization devices (4.5 mm × 35 mm/5 mm × 35 mm/5 mm × 30 mm) telescoped, DSA showed blood flow retention in the aneurysm, and no abnormalities were found in the branches or distal vessels. **(C)** Complete occlusion was seen at the 3-month DSA follow-up examination.

For fusiform aneurysms with a large range, it is difficult for a PED to cover the entire lesion, or even if it can cover the neck of the aneurysm, anchoring of the PED in the parent artery is unstable, and stent displacement may occur during or after the procedure. Griffin retrospectively reviewed consecutive patients treated with PEDs for fusiform aneurysms. On DSA follow-up, only 15 aneurysms (60%) were completely occluded, which was a relatively lower rate than that for saccular aneurysms (Griffin et al., 2021). Conversely, our study showed satisfactory outcomes, with 85.7% (12/14) of fusiform aneurysms shown to be occluded using multi-PEDs. Furthermore, this can be achieved within 3 months if the coil fills the neck and if multi-PEDs are telescoped for reconstruction (Figure 1). Unfortunately, such aneurysms are more common in the posterior circulation. A total of two of the 14 patients in our study experienced severe ischemic complications, leading to post-operative death. This was in line with Griessenauer who reported that the complication rate of PED use in the basilar artery was significantly higher than that for PED use in the anterior circulation, particularly in the incidence of multiple stent ischemia complications, which is in the range of 50–70% (Griessenauer et al., 2018). Given the above, the application of multi-PEDs is not suitable for large-scale basilar artery or aneurysm-like lesions. For such lesions, if PED implantation is the only choice, it is better to cover the entire lesion with one PED or a long low profile self-expandable (LEO) stent, followed by one PED to cover the lesion within the LEO stent; otherwise, the strategy should be changed.

**FIGURE 2:**
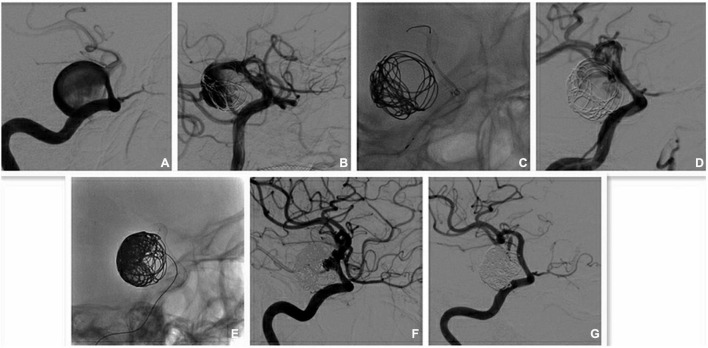
Multi-PEDs for the treatment of a giant aneurysm (overlap technique). **(A)** Digital subtraction angiography (DSA) identified a large aneurysm with jet-sign at the narrow neck in the internal carotid artery (ICA) before operation. The proximal diameter and distal diameter were 4.55 and 3.77 mm, respectively. **(B)** The jet-sign is decreased slightly after the implantation of a pipeline embolization device (PED; 4.25 mm × 35 mm) and coil embolization. **(C)** The second PED (4.5 mm × 18 mm) was overlapped within the first one to cover the neck. **(D)** The ejection (jet-sign) is decreased largely after the implantation of two PEDs and coil embolization. **(E)** More coil was used to fill the entrance area of the sac. **(F)** DSA shows that the aneurysm was nearly occluded a week after operation. **(G)** The aneurysm is completely occluded, and the patency of the parent arteries is satisfactory at the 3-month DSA follow-up examination.

**FIGURE 3:**
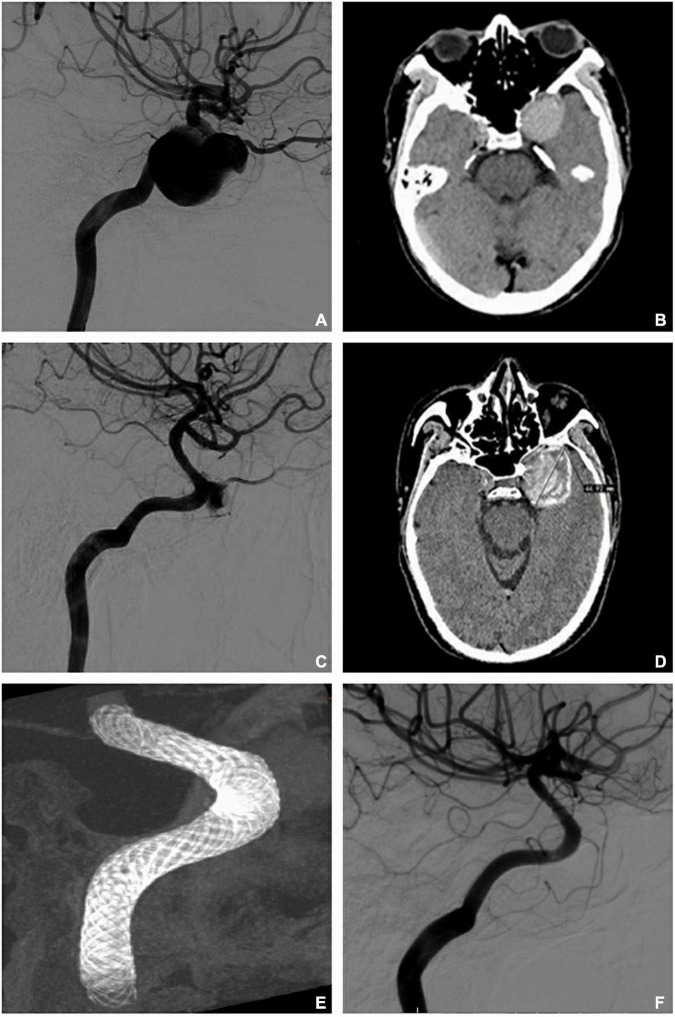
Retreatment for unoccluded aneurysms (telescope and overlap techniques). **(A)** Digital subtraction artery (DSA) identified a giant aneurysm with a proximal diameter of 4.33 mm and a distal diameter of 3.93 mm in the internal carotid artery before operation. **(B)** Computed tomography (CT) revealed the maximum diameter of the aneurysm as 32 mm before operation. **(C)** DSA showed no occlusion after the implantation of two telescoped pipeline embolization devices (PEDs; 4.75 × 35 mm/4.75 × 30 mm) for 36 months. **(D)** CT shows that the sac enlarged over 36 months (maximum diameter = 45 mm). **(E)** Vaso-CT demonstrates the reconstruction after another two PEDs (4.5 mm × 30 mm/4.5 mm × 20 mm) overlapped at the neck of the aneurysm. **(F)** Complete occlusion is seen at 10 months after retreatment with the two overlapping PEDs.

The PUFS trial first demonstrated the safety and effectiveness of multi-PEDs in the treatment of large or giant intracranial aneurysms. The mean size in this study was 18.2 mm and the majority of patients (98.1%) were treated with more than one PED (average of 3.15 PEDs). At 1 year, angiography showed complete occlusion in 76 of the 96 patients (86.8%) (Becske et al., 2013). In Japan, Oishi used PED to treat 100 large/giant aneurysms of the internal carotid artery, and each patient used 1.42 PEDs on average. Follow-up angiography showed an occlusion rate of 69.2% at 1 year (Oishi et al., 2018). These two studies showed quite different occlusion rates, which may be caused by the large number of PEDs used in PUFS. This finding was consistent with the results of our study. In our study, 49 aneurysms (≥ 10 mm) were implanted with 108 PEDs in total. For each patient in the study, 2.2 PEDs were used on average and the occlusion rate was 83.3% (25/30). Furthermore, these large/giant arteries were more common in the outer curve of the parent artery. Niimi applied Doppler technology to measure the blood flow velocity of an extracorporeal aneurysm model and proposed that the velocity and rupture risk of the aneurysm in outer curved vessels were higher than those in inner curved vessels (Niimi et al., 1984). Meng conducted a comparative study on low-curvature and high-curvature aneurysms with stent implantation, using dog and rabbit aneurysm models, and found that, with the increase in the parent artery curvature, the influence of stents on aneurysm hemodynamics decreased (Meng et al., 2014). Therefore, the usual solution strategy was to compact or overlap the stents. Damiano conducted a computational study and reported that the effect of a compact PED was less than that of two overlapping PEDs in saccular aneurysms (Damiano et al., 2017). However, Chalouhi stated that the use of multiple stents increased the incidence of ischemia-related symptoms (Chalouhi et al., 2014). In our study, only one patient with anterior circulation developed severe nerve defects (mRS 4), but recovered rapidly. Another patient had a large aneurysm with a jet sign at the neck. We attempted to use multi-PEDs to enhance the diversion effect and achieved positive results as seen at the 3-month DSA follow-up examination (Figure 2). In addition, a patient received two telescoped PEDs, without coil embolization, because of the large difference in diameter between the outflow and inflow vessels. However, the aneurysm was found to be unoccluded, and the sac enlarged, after 36 months. The aneurysm was eventually occluded by 10 months, after the application of another two overlapped PEDs in retreatment (Figure 3). It can be concluded that overlapping PEDs are suitable for use in large/giant aneurysms, particularly when there is a jet-sign after PED implantation. As described, the use of multi-PEDs has a certain safety and efficacy in the anterior circulation. For the posterior circulation lesions, due to the large number of perforating arteries, strict evaluation and further study remain indispensable.

Poor wall attachment plays a significant role in the treatment of complex lesions, including immediate and delayed stent displacement or retraction (Brinjikji et al., 2016; Heit et al., 2017). When this occurs, salvage of failed PED treatment is necessary. In our study, there were five patients with proximal retraction into the sac and poor adhesion during implantation. In these cases, we chose to deploy a second PED. Follow-up demonstrated that the aneurysms were completely occluded, patency of the parent arteries was satisfactory, and there were no related complications. Thus, for aneurysms with very tortuous parent arteries, acute angles between inflow and outflow vessels, or greatly varying diameters, telescoping short and small PEDs are also an option to avoid poor opening or wall attachment during operation.

In addition, the application of a single PED assisted with coil embolization in the treatment of ruptured intracranial aneurysms has also been reported, and the short-term occlusion rate and incidence of complications are acceptable. Although the risks were higher than those of unruptured aneurysms, all occurred during the perioperative period (Chalouhi et al., 2015; Lin et al., 2015; Mokin et al., 2018). Nevertheless, there remains a 16% risk of aneurysm recurrence and re-rupture, particularly for blood blister-like aneurysms (Ji et al., 2017; Zhu et al., 2018). Thus, a multi-PED technique might be a promising option for this type of aneurysm.

### Operation Strategies and Skills of Multiple Pipeline Embolization Devices

Strategies for using multi-PEDs include telescoping and overlapping techniques, with or without coil embolization. The detailed operations are as follows:

For telescoping, the first point is that the selection of the longest length and diameter should be larger than the anchoring zone. The first PED should be anchored firmly and for a sufficient length in the outflow vessel to ensure stability. Thus, the first PED achieves good opening and wall apposition. It should not be displaced when the second PED is pushed and pulled within the first PED. The diameter of the second PED should not be smaller than that of the first. When releasing the second PED, our experience is that it is necessary to ensure that the distal part of the second PED should overlap the first PED by 1/2 to 1/3 of its length. Thus, the second PED can cross the neck of the aneurysm safely along the small curve and be anchored stably in the proximal normal inflow vessel. If the system is not anchored stably, the third PED must be considered in the previous steps. As for the release, the first PED is implanted from distal to proximal and is released *in situ* or in slightly distant vessels. In other words, the distal part of the PED should be anchored in the normal outflow vessels with sufficient length, and the proximal part is then released into the inflow vessel or sac. When releasing the second PED, the stent catheter passes through the first deployed PED along the middle support wire of the conveying stent and is then deployed. The release of the second PED is mostly *in situ*, within the former PED, and a push release is mostly used. Once the PEDs cover the whole neck and after the final release of the last PED, intra-arterial rotational angiography (VasoCT, Philips Healthcare) or dynamic CT angiography (Dyna CT, Siemens Healthcare) is performed to identify whether the PEDs are opened well and have achieved good wall attachment, before coil embolization; otherwise, post-operative adjustment with guidewire and balloon application is the next step. To increase the stability of the multi-PEDs for patients with large/giant aneurysms, the embolization microcatheter is prepositioned in the sac, in parallel with the stent catheter in a 7F guiding sheath, in advance. After angiography confirms that the PED system completely covers the neck, the sac is filled with the coil to support the stability of the PEDs and promote thrombosis (Bender et al., 2018). Generally, a size larger than the diameter of the sac is selected, although the packing density is not crucial. More contact between the coil and PED at the neck is important for achieving better support and diversion. If the sac does not support the coil, the PED is pushed against the large curve, facilitating release. Simultaneously, concurrent insertion of more PEDs is possible.

The overlapping of PEDs is not complicated, and the operation is similar to that of a normal stent. Because the PED is fully visible, the proximal and distal positions and the opening and wall attachment can be clearly recognized under fluoroscopy or angiography. The overlapping technique entails deployment of a longer and a shorter PED. The longer stent is deployed first. It should be noted that the first PED must be as long as possible to cover the neck and be stably placed, so that the PED catheter can pass through it safely. The second PED is shorter (covering only the neck), but the diameter is equal to that of the first PED. The shorter stent is deployed within the lumen of the longer stent.

There are several challenges in the implantation of a PED. When multi-PEDs are implanted, there are more risks. One of the most difficult situations is the loss of the distal outflow vessel after the first PED is released. At this point, the only option is to use a microcatheter and microguidewire to find the back outflow vessel using biplane fluoroscopy. A somewhat soft and thin microcatheter can be selected to reach far from the outflow vessel and is then exchanged for a slightly harder microguidewire or catheter. Alternatively, the stent catheter or intermediate catheter can be introduced after anchoring with a balloon or stent. Moreover, poor opening and wall attachment may occur when the path is tortuous. In such a case, a balloon or balloon-expanding stent can be applied when the multi-PED system is stable. Otherwise, retreatment is an option in the second stage. For complications of thrombosis or microembolism, a moderate dose of tirofiban can be administered intraoperatively in addition to dual antiplatelet therapy.

This study has potential limitations. First, this is a retrospective observational study based on a small sample size. There are therefore subject to biases and confounding that may have influenced our results. Second, clinical results may be influenced by the differences in treatment strategies and experiences at different centers. Third, much longer follow-up and larger sample sizes need to be used to assess the effectiveness of multi-PED implantation. In addition, multicenter prospective studies need to be designed in the future for the study.

## Conclusion

The use of multi-PEDs is safe and effective for the treatment of aneurysms in the anterior circulation, but is not recommended for those in the posterior circulation. Implantation of multi-PEDs could be considered for large-scale fusiform aneurysms, large/giant saccular aneurysms with a jet-sign after PED implantation, as salvage for failed PED treatment, and in cases where the diameter of the parent artery varies greatly.

## Data Availability Statement

The raw data supporting the conclusions of this article will be made available by the authors, without undue reservation.

## Ethics Statement

The study was approved by the Ethics Committee of The First Affiliated Hospital of Zhengzhou University (KY 2018-098-02). The patients/participants provided their written informed consent to participate in this study.

## Author Contributions

FF carried out the studies, participated in collecting data, and drafted the manuscript. SG performed the statistical analysis and participated in its design. All authors contributed to the article and approved the submitted version.

## Conflict of Interest

The authors declare that the research was conducted in the absence of any commercial or financial relationships that could be construed as a potential conflict of interest.

## Publisher’s Note

All claims expressed in this article are solely those of the authors and do not necessarily represent those of their affiliated organizations, or those of the publisher, the editors and the reviewers. Any product that may be evaluated in this article, or claim that may be made by its manufacturer, is not guaranteed or endorsed by the publisher.
